# Resident perception on the impact of point-of-care ultrasound in clinical care at a family medicine training program in Zambia

**DOI:** 10.1186/s13089-022-00273-7

**Published:** 2022-05-15

**Authors:** Matthew S. Haldeman, Evaristo Kunka, Mpundu Makasa, Bassim Birkland

**Affiliations:** 1 Department of Community and Family Medicine, School of Public Health, University of Zambia, P.O. Box 50110, Lusaka, Zambia; 2 Seed Global Health, 20 Ashburton Place, Boston, MA 02108 USA

**Keywords:** Point-of-care ultrasound, Family medicine, Zambia

## Abstract

**Background:**

Patient access to necessary medical imaging in low- and middle-income countries (LMICs) remains a major obstacle, complicating clinician decision-making and compromising patient outcomes.

**Methods:**

We implemented a longitudinal point-of-care ultrasound (POCUS) training program at a new Family Medicine residency in Zambia and subsequently evaluated residents’ perceptions on the impact of POCUS in patient care. Data were documented by the scanning resident via a post-scan survey, which assessed if/how the scan assisted in medical management, and if/how the scan changed that management. The primary endpoint was frequency of scans assisting and changing management. Data were summarized using descriptive statistics.

**Results:**

Over the 1-year study period, 366 patient encounters occurred in which POCUS was utilized, resulting in a total of 542 unique POCUS scans. POCUS assisted in decision-making in 95.6% (350/366) of patient encounters, most commonly by helping to determine a diagnosis. POCUS changed management in 65.8% (235/357) of patient encounters, most commonly leading to a medication change.

**Conclusions:**

Zambian resident physicians perceived POCUS to be very helpful in their clinical decision-making. These data support the need to advance POCUS education at the residency level throughout LMICs, which may be an ideal strategy to promote widespread utilization of POCUS in low-resource settings globally.

## Background

Throughout low- and middle-income countries (LMICs), patient access to necessary medical imaging remains a major obstacle, complicating clinician medical decision-making and potentially resulting in poorer patient outcomes [10, 23]. Availability of imaging modalities, such as X-ray and diagnostic ultrasound, is often limited by a number of factors, including cost, lack of regular maintenance, power outages, lack of technical expertise, exhausted supply of required materials (such as X-ray films), and limited portability to reach unstable patients. Access to more advanced imaging modalities, such as computed tomography (CT) and magnetic resonance imaging (MRI), is usually even further restricted, if available at all [10, 23]. Sub-Saharan Africa is no exception to these limitations, where clinicians are often forced to make medical decisions with limited diagnostic data [10].

Point-of-care ultrasound (POCUS) has been shown to reduce procedural complications, enhance diagnostic accuracy beyond the physical exam, and expedite definitive care [19]. While POCUS has had significant impact in high-resource settings, its potential impact in LMICs is enormous [2, 25]. Given its portability, affordability, excellent safety profile, low maintenance requirements, and ability to operate independent of consumable resources, POCUS may be an ideal imaging modality in these settings [2, 24, 25].

Both the utilization of—and clinician training in—POCUS has increased in LMICs in recent years [2, 7, 22, 24]. Multiple studies have demonstrated the positive impact of POCUS on clinician decision-making and patient outcomes in these regions [10, 19, 22, 23]. Reynolds et al. [19] collected data on all POCUS scans performed in a busy Emergency Department setting in Dar es Salaam, Tanzania over a 10-month period, using pre/post clinician surveys to estimate impact of POCUS on clinical decision-making. Investigators found that POCUS changed clinical management in 29% of cases, but this number increased to 45% when multiple POCUS scans were performed [19]. Despite the ample evidence of POCUS’s impact in LMICs, POCUS training for most clinicians in these regions—if available at all—is often achieved via truncated training sessions by short-term visiting clinicians without long-term follow-up or quality control [25]. Adequate POCUS training at the residency level in LMICs is still uncommon due to lack of resources, technical expertise, and mentorship availability, among other reasons [21].

The University of Zambia (UNZA) launched Zambia's first Family Medicine residency program in 2019, which spans 4 years and leads to the award of a Master’s of Medicine (MMed) degree. Through a partnership with the U.S.-based non-profit organization Seed Global Health, the program’s first faculty arrived in early 2020. Seeing a critical need for diagnostic medical imaging in this low-resource setting, the faculty performed an initial POCUS needs assessment among all UNZA Family Medicine clinicians and subsequently integrated a longitudinal POCUS training course into the larger residency curriculum.

The healthcare system in Zambia is government-run but decentralized, comprised of first-level facilities (health posts, health centers, and district-level hospitals), second-level facilities (provincial hospitals), and tertiary-level facilities (tertiary teaching hospitals) [27]. Basic healthcare services are offered free-of-charge, but additional services are offered at a cost to the patient [18]. The country is committed to universal healthcare coverage, but significant healthcare inequities still exist [18, 26]. Chilenje Level 1 Hospital, a 92-bed entry-level district hospital in Lusaka, serves as UNZA Family Medicine’s primary clinical training site. Chilenje serves a catchment population of > 130,000, comprised of patients from all income levels [15]. Laboratory services at the hospital are limited, with only basic labs—hemoglobin, glucose, malaria, and HIV, among a few others—available on-site, all other lab samples are sent to outside facilities at a cost to the patient. While the hospital does offer X-ray and ultrasound services to its patients, these imaging services require patient payment, are often unavailable due to maintenance issues, lack portability to reach unstable patients, and are limited in scope (echocardiography and lower extremity deep venous thrombosis (DVT scanning are not performed, for example. Given the hospital’s high patient burden of conditions that benefit from imaging—specifically respiratory infections (including tuberculosis), hypertension (with its cardiovascular sequelae) and trauma [15]—the patient imaging needs are great and often go unmet. The combination of these factors made Chilenje hospital an ideal site for implementation of a POCUS program. In addition, given its flexibility as a new residency program and its access to Chilenje’s diverse patient population, UNZA Family Medicine presented an ideal residency framework for such a study. The purpose of this study was to evaluate residents’ perceptions of POCUS impact on medical decision-making at a new Family Medicine residency program in Zambia.

## Methods

This was a single-center, survey-based, prospective study conducted with UNZA Family Medicine resident physicians. All survey data were collected at the program’s primary clinical training site, Chilenje Level 1 Hospital in Lusaka. POCUS workshops occurred primarily in the classrooms of UNZA’s Ridgeway campus. This study was reviewed by the institutional review board (IRB) at UNZA Biomedical Research Ethics Committee (UNZAREC) as well as the National Health Research Authority (NHRA) of Zambia. It was deemed exempt from full review by both regulatory bodies, and permission was granted to conduct the study by the hospital administration. The study’s participants included all residents enrolled in the UNZA Family Medicine program between 05/2020–05/2021.

POCUS education was implemented by Family Medicine faculty who had previously undergone fellowship-level POCUS training. All ultrasound education and patient scanning was performed with three Butterfly iQ™ (Butterfly Network, Guilford, CT, USA) handheld ultrasound devices. The device’s manufacturer, Butterfly network, partnered with UNZA Family Medicine by waiving device annual subscription fees and donating oneButterfly iQ™ device to the program.

All resident physicians, regardless of year, were enrolled in the POCUS training simultaneously, but participation in the study was voluntary. None of the residents had any previous POCUS experience prior to this course. POCUS education was accomplished via monthly workshops, which each lasted approximately 2–3 h and consisted of didactics followed by hands-on skills practice with patient models. Workshops occurred over an 11-month period beginning February, 2020. Topics of monthly workshops included an introduction to POCUS, lower extremity deep venous thrombosis (DVT) assessment, abdominal aortic aneurysm (AAA) and inferior vena cava (IVC) assessment, liver/gallbladder assessment, basic pulmonary ultrasound, basic obstetric ultrasound, renal/urinary bladder assessment, basic echocardiography (divided into 2 workshops), ultrasound in the undifferentiated shock patient (“Rapid Ultrasound for Shock and Hypotension” or “RUSH” protocol [17]) and ultrasound assessment of the respiratory failure patient (“Bedside Lung Ultrasound in Emergency” or “BLUE” protocol [12, 13]). In addition to workshops, supervised scanning opportunities for residents were available during inpatient rounds, clinic, and at dedicated ultrasound rounds. Residents created scan portfolios by uploading their images into individualized folders onto the Butterfly Network™ cloud and also participated in periodic image review sessions with faculty. For those completing the program, a POCUS final exam in the form of an Objective Structured Clinical Examination (OSCE) was administered, which included both a written exam and assessment of POCUS skills at the bedside. Given the study’s initiation in May, 2020, all participating residents completed the first 3 workshops prior to the study’s initiation. While the study overlapped with remainder of the POCUS course, residents only performed scan types in the clinical setting with which they were familiar.

Patients were selected for POCUS scanning based on clinical necessity—specifically diagnostic queries in need of clarification, or procedures in need of imaging guidance—as determined by the rounding clinical team (inpatient) or the participating resident and faculty (outpatient). In addition, a patient’s ability to obtain alternative imaging on his own was taken into account; if there existed viable standard imaging options which the patient could afford and obtain in a reasonable time, POCUS was deferred in favor of those options. If a patient identified for POCUS in any of these settings was discovered to have already undergone radiology-performed ultrasound scanning, POCUS was deemed unnecessary unless the team had outstanding questions, in which case POCUS was still performed. Patients meeting these eligibility criteria for POCUS scanning were informed and verbal consent was obtained per hospital protocol, and scanning was performed by a resident physician with faculty supervision.

Given the study’s probes remained with faculty at all times, and faculty schedules were limited, the ultrasound probes were available for patient scanning approximately 4 half-days per week, which included teaching inpatient rounds (two half-days per week), clinic (one half-day per week), and dedicated scanning rounds (one half-day per week). Scanning was not regularly performed in the Emergency Department (ED) or on Labor and Delivery (L&D) due to faculty schedule limitations, but consults for a scan from these departments were completed as schedules allowed. All scans were performed with faculty present, and all images were immediately interpreted for timely application to patient care. Feedback was also given at this time to the scanning resident. Initially, all scans were performed with direct faculty supervision, but over the course of the POCUS training, some residents put forth extra time and effort and were able to demonstrate adequate image acquisition skills for particular scan types. If a resident could acquire adequate images without assistance in at least 3 supervised scans, that resident would be permitted to perform image acquisition for those scans independently on rounds or in clinic with faculty nearby, while still requiring immediate subsequent faculty image review for interpretation and application to patient care. Residents were not permitted to perform independent scans when no faculty were available. Given some scan types are more complex to perform than others (e.g., cardiac), residents would, on average, have to perform more of those scans to demonstrate 3 adequate ones than they would for simpler scan types. At any time, if resident-obtained images were found to be inadequate, faculty would immediately assist the resident with appropriate image acquisition and further supervision would still be required. Thus, all POCUS images were reviewed by faculty to ensure quality image interpretation and optimal application to patient care.

**Fig. 1:**
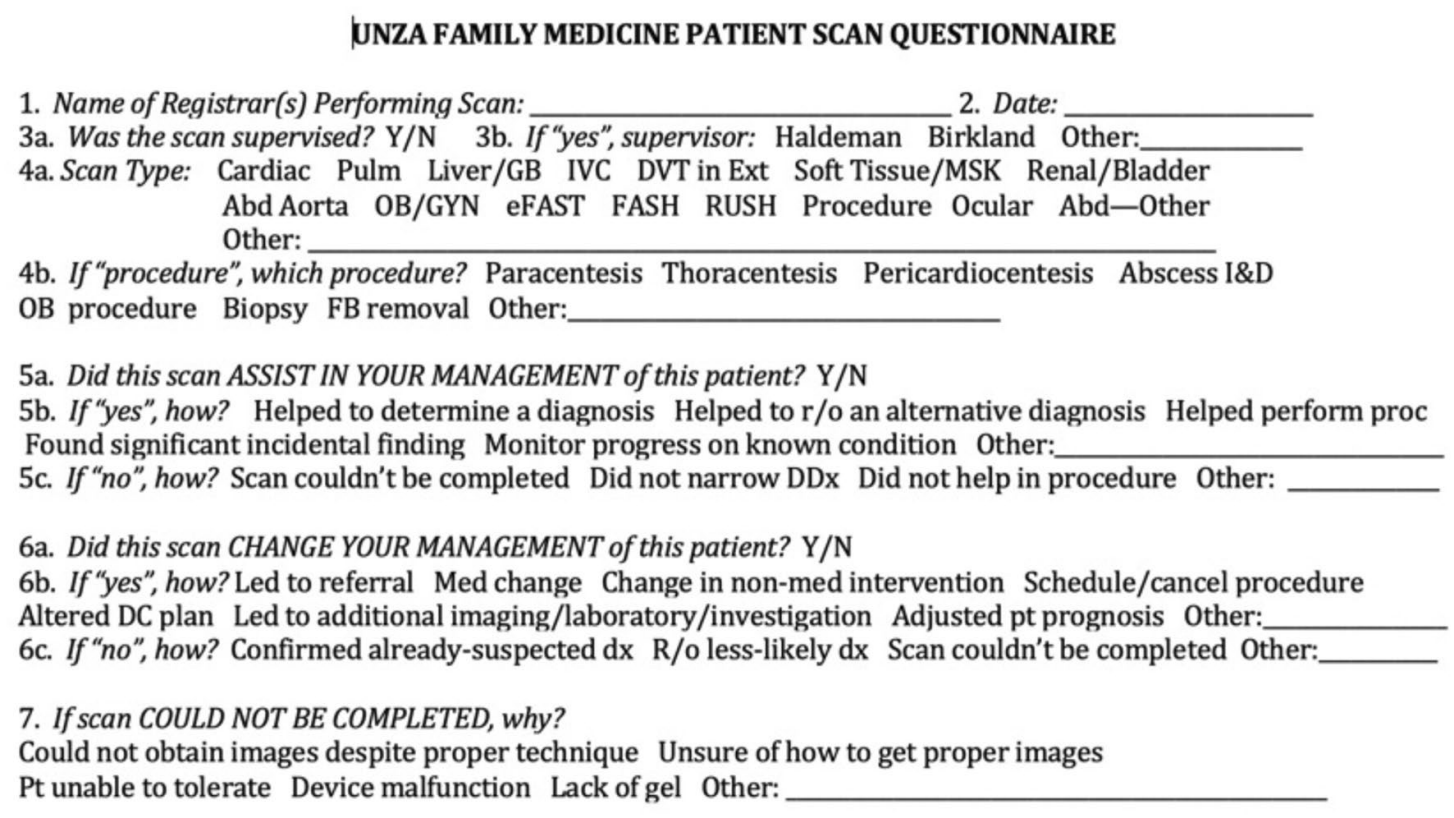
Patient scan questionnaire

Resident’s perception of ultrasound scan impact on clinical decision-making was estimated via a “Patient Scan Questionnaire” (Fig. 1), which the scanning resident completed after each patient scan was performed. Patient scans (and the subsequent surveys) were distributed evenly on a rotating basis among residents rotating on a given service. These surveys were collected for all resident scans for a period of 1 year, from May 5, 2020 through May 4, 2021. The Patient Scan Questionnaire recorded resident (registrar) name, date, type of scan, presence/absence of supervision, if/how the scan assisted with medical management, and if/how it changed medical management. If a scan could not be completed, this was noted and the reason was recorded. If more than one resident was present during scanning, all would be engaged in the survey discussion but the scanning resident’s final opinion was the one recorded. If a resident desired to give a response that was not one of the options, an “Other” category was available and the response was written in. Residents completed the patient scan questionnaires on their own, without faculty surveillance. No surveys were completed for scans that were performed purely for resident practice and did not apply to patient care. No patient-identifying information was recorded at any point during the study in any format.

Data were collated in Microsoft Excel for Mac 2021 (version 16.50). Statistical analysis was performed using descriptive statistics. Primary outcomes included percentage of scans that assisted in management and percentage of scans that changed management. Secondary outcomes included numbers and types of scans performed, distribution of how scans assisted or did not assist with management, distribution of how scans changed or did not change management, percentage of scans that assisted with management by scan type, percentage of scans that changed management by scan type, and distribution of why scans could not be performed.

## Results

**Fig. 2:**
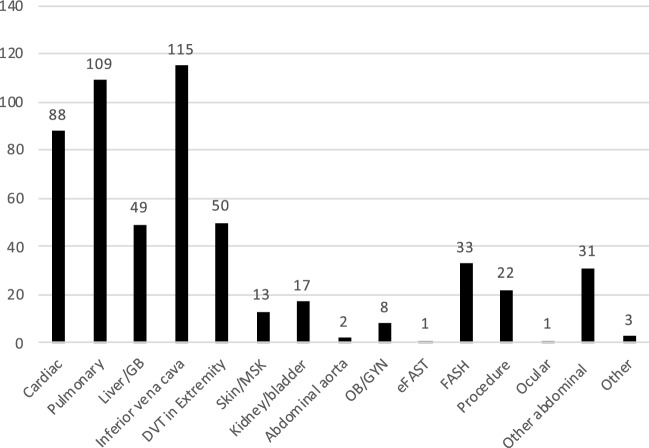
Scan types performed. DVT: deep venous thrombosis; GB: gallbladder; FASH: Focused Assessment with Sonography for HIV-associated tuberculosis; MSK: musculoskeletal; OB/GYN: Obstetrics and Gynecology; eFAST: Extended Focused Assessment with Sonography in Trauma

Over the 1-year study period, 10 resident physicians (7 in 1st year, one in 2nd year, and two in 3rd year) underwent the longitudinal POCUS training and all agreed to participate in the study. None had any previous POCUS experience. There were 366 patient encounters in which POCUS was utilized in clinical decision-making, resulting in a total of 542 unique POCUS scans. Two residents showed great diligence and achieved independent image acquisition skills for all scan types. These two residents contributed to a large number of patient POCUS encounters (combined total of 266, out of the 366 patient encounters), while the remaining patient encounters were distributed relatively evenly among the other 8 residents. The most frequently performed scan type was IVC assessment, which consisted of 21% (115/542) of scans. The remaining scan types and their frequencies are presented in Fig. 2. The majority of patient scans (76.8%, 281/366) were supervised by faculty. Of note, while the Patient Scan Questionnaire listed the Rapid Ultrasound for Shock and Hypotension (RUSH) protocol as a scan type, in practice this was separated into cardiac, eFAST, and IVC scan types.

A total of 22 ultrasound-guided procedures were performed, of which 13 were combined with other diagnostic scans and 9 were procedure-only scans. The most frequent procedures performed were paracentesis (14), thoracentesis (5), and “other” (3), which included 1 knee injection, 1 suprapubic bladder aspiration, and 1 internal jugular central line placement.

**Fig. 3:**
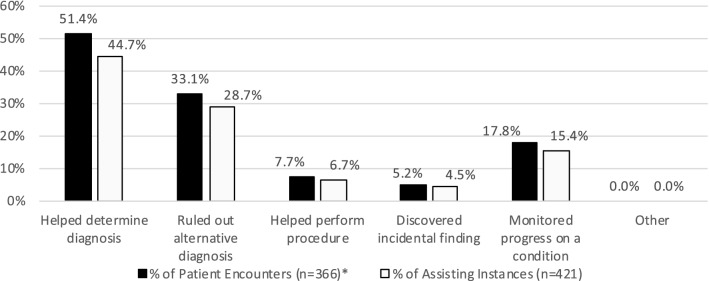
Distribution of assist instances. *Because the majority of scans assisted management, and scans often assisted in > 1 way, the sum of percentages for patient encounters is > 100%

Scans assisted in clinical management in 95.6% (350/366) of patient encounters, and since scans often assisted in > 1 way, for a total of 421 assisting instances. POCUS assisted most frequently by helping to determine a diagnosis, which it did in 51.4% (188/366) of patient encounters and in 44.7% (188/421) of assisting instances. The remaining assisting frequencies are presented in Fig. 3.

When using POCUS for procedural guidance alone, residents were confused when asked if POCUS changed their clinical management, given procedural POCUS was generally non-diagnostic and meant only to assist. After discussion, it was decided that the “changed management” question did not apply for procedure-only scans, and instead a “not applicable” (“N/A”) option was created and selected for these instances. A total of 9 patient encounters involved procedure-only scans, which were subtracted from the total patient encounters for this question, reducing its total to 357. This was not applied to patient encounters which involved both diagnostic and procedural scans.

**Fig. 4:**
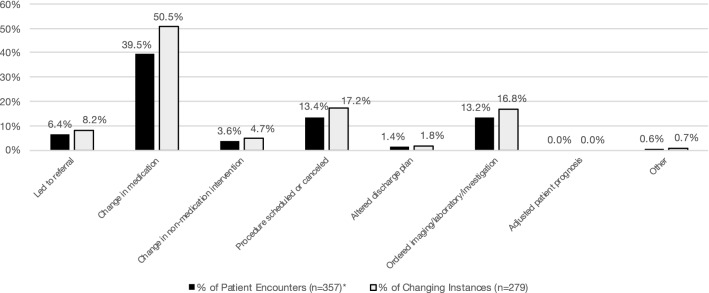
Distribution of changing instances. *Because not all scans changed management, the sum of percentages for patient encounters is < 100%

**Fig. 5:**
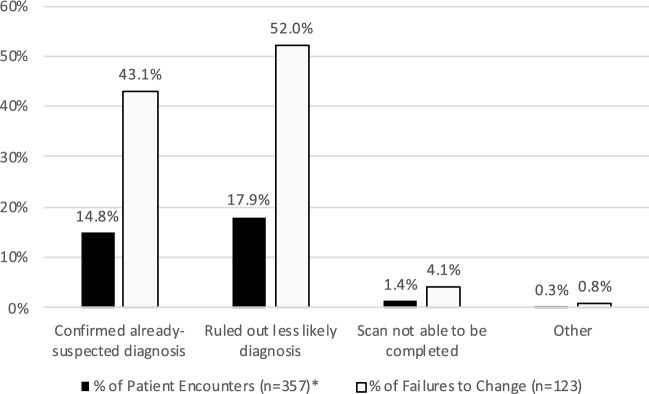
Distribution of absence of change instances. *Because only a minority of scans resulted in absence of change, the sum of percentages for patient encounters is < 100%

Scans changed clinical management in 65.8% (235/357) of patient encounters, and since scans often changed management in > 1 way, a total of 279 changing instances occurred. POCUS changed management most frequently by leading to a medication change, which it did in 39.5% (141/357) of patient encounters and 50.5% (141/279) of changing instances. The remaining changing frequencies are presented in Fig. 4. If a scan did not change management, this was categorized as an “absence of change,” which occurred in 34.2% (122/357) of patient encounters. The most common reason for absence of change was that the scan ruled out a diagnosis that was already deemed unlikely, which occurred in 17.9% (64/357) of patient encounters and in 52.0% (64/123) of absence of change instances. The remaining absence of change frequencies are presented in Fig. 5. Of note, 5 absence of change instances were due to the scans not able to be completed, which was further investigated by survey question #7. In 3 of these 5 scans, images could not be obtained despite proper technique, and in the remaining 2 scans, the patients did not tolerate scanning. Table 1 displays the assisting and changing frequencies of POCUS scans by scan type.

**Table 1:** Scan impact by scan type

Scan type	# Performed	Assisted (#)	Assisted (%)	Changed (#)	Changed (%)
Inferior vena cava	115	113	98.3%	88	76.5%
Pulmonary	109	107	98.2%	73	67.0%
Cardiac	88	86	97.7%	61	69.3%
DVT in Extremity	50	49	98.0%	31	62.0%
Liver/GB	49	46	93.9%	32	65.3%
FASH	33	26	78.8%	23	69.7%
Other abdominal	31	29	93.5%	26	83.9%
Procedure*	22	22	100.0%	N/A	N/A
Kidney/bladder	17	16	94.1%	13	76.5%
Skin/MSK	13	13	100.0%	9	69.2%
OB/GYN	8	8	100.0%	5	62.5%
Other	3	3	100.0%	3	100.0%
Abdominal aorta	2	2	100.0%	2	100.0%
eFAST	1	1	100.0%	1	100.0%
Ocular	1	1	100.0%	1	100.0%
Total Unique Scans	542				

^*^Given a portion of the POCUS procedures were procedure-only, which received an “N/A” answer for survey question #6, change frequencies and percentages were unable to be calculated, and the “N/A” was carried through into this table. DVT: deep venous thrombosis; GB: gallbladder; FASH: Focused Assessment with Sonography for HIV-associated tuberculosis; MSK: musculoskeletal; OB/GYN: Obstetrics and gynecology, eFAST: Extended Focused Assessment with Sonography in Trauma

## Discussion

We performed a survey-based evaluation of Family Medicine residents’ perceptions of POCUS impact on medical decision-making in a Level 1 Hospital in Zambia. We found their perceptions of this impact to be large for the patients that they scanned.

POCUS assisted and changed management in the majority of patient encounters. POCUS was found to assist medical management almost universally, likely due to the fact that both positive and negative scan findings often assisted the clinical team’s decision-making. POCUS was also found to change medical management in approximately 2/3 of patient encounters, likely a reflection of the relative unavailability of alternative imaging options and other diagnostics (such as most laboratory tests) for many patients at our study site. Therefore, for the vast majority of patient who received POCUS exams, residents perceived POCUS to be impactful.

Although we did not ask residents to record specific diagnoses or scanning indications in the Patient Scan Questionnaire, anecdotally residents reported that IVC, pulmonary, cardiac, and FASH (Focused Assessment with Sonography for HIV-associated tuberculosis) scans were found to be very helpful [6, 8]. IVC scanning assisted frequently in estimating central venous pressure (CVP) and fluid tolerance for shock patients, and when combined with pulmonary scanning, assisted in the evaluation of patients with undifferentiated respiratory failure. Residents reported that both conditions were commonly encountered, hence the high numbers of IVC scans performed in this study. Given the hospital’s X-ray machine was frequently non-functional, residents reported that pulmonary ultrasound proved useful in the evaluation of respiratory patients and management of pleural effusions—which are commonly encountered in this setting. Finally, they found cardiac echo useful in assessment of patients with likely congestive heart failure, which is underdiagnosed in our setting, and FASH scanning empowered them to make a presumptive diagnosis of extra-pulmonary tuberculosis, which can be difficult to diagnose definitively.

The potential impact of POCUS on patient outcomes in LMICs is tremendous [2, 25]. While a variety of POCUS education methods have been implemented in LMICs previously, the findings from this study suggest that integration of POCUS education directly into residency training curricula in LMICs is both feasible and perceived by resident physicians as having large impact in their clinical care. Indeed, POCUS education at the residency level may prove to be a useful tool for POCUS to be more broadly adopted—and regularly utilized—by clinicians practicing in LMICs. In addition, expanded availability of handheld devices with whole-body scan abilities, low power requirements, and ultra-portability will likely further support the utilization of POCUS in these settings [2].

Integrating POCUS education at the residency level in LMICs has multiple benefits. First, clinicians in residency are still developing their medical skills, allowing them to embrace POCUS and incorporate it into their patient care more easily than seasoned clinicians. Second, residency is a formative time, when clinicians develop many habits that persist long-term. By incorporating POCUS into their residency training, resident clinicians will more likely incorporate POCUS into their practice and utilize it throughout their medical careers—potentially impacting the outcomes of thousands of future patients [5, 21]. Even if these clinicians cannot afford to purchase their own handheld devices, many will have access to an ultrasound machine of some sort at their future clinical sites. Finally, incorporating POCUS education at the residency level allows for the possibility of longitudinal POCUS training with direct supervision and ample opportunities for hands-on practice—features which are often unavailable in short-term POCUS trainings [21].

How can POCUS training be effectively and sustainably integrated into LMIC residency programs? One option includes the sending of POCUS-trained physicians to serve as visiting faculty at such residency programs for a longer period (e.g., 1 year or more), which allows for longitudinal training of both residents and local faculty. Ideally, interested local faculty can also be trained as “POCUS champions” and serve as POCUS educators for their own programs in the future—a “train the trainers” model [1, 4]. An alternative is longitudinal remote POCUS education, involving both online didactics and remote mentorship sessions spread over a longer time (months to > 1 year). Online platforms enhancing effective remote mentorship are already available. Finally, shorter term options such as a POCUS away rotation or a short-term workshop are possible but would likely have greater impact if paired with some form of long-term POCUS mentorship. Whatever the method, the features of an optimal POCUS educational program in LMICs would include longitudinal duration, direct supervision, opportunities for hands-on practice, and long-term mentorship—similar to what is most effective in high-resource settings [3, 9].

### Limitations

There are several limitations to this study. First, the survey data contained both subjective and objective data points. Survey items #5a and #6a, which ask if the scan assisted or changed management, respectively, are somewhat subjective. Improved survey item design to make the survey more objective would be beneficial for future studies. In addition, the potential for bias is significant for several reasons. First, the survey was administered to residents only after the scans were performed, without baseline data present. A more objective attempt at quantifying POCUS’s impact, such as comparing the differential diagnosis and/or the patient plan using surveys both pre- and post-scan, may serve to eliminate some of this bias. Also, a resident’s pre-existing clinical acumen may influence the degree to which she views POCUS as having assisted her management, and this was not accounted for in this study. Controlling for residents’ training levels may strengthen future study. Next, the questionnaires were not anonymous, potentially adding additional bias to the survey results. Finally, the study’s design places it at significant risk for reporting bias, given visiting faculty served to supervise the residents’ scans, assist in image interpretation, and subsequently administer the survey to the same residents. Those residents may have felt pressure to answer survey questions more positively that they would have otherwise, possibly inflating the study’s results. Ideally similar surveys in future studies would be performed by an alternative non-faculty data collector.

The study used surrogate markers of patient-level impact, not direct measures. Patient-level indicators, such as mortality, complication rates, additional procedures, etc., were not directly recorded, which limits the study’s applicability to patient care. Ideally future studies would involve chart review or other means of tracking patient-level impact. In addition, patients were selected to be scanned based off of clinical necessity, as determined by the clinical team—which can be a subjective process at times. This could potentially open the data to selection bias. The study’s sample size was relatively small (*n* = 366 patients scanned), which was mostly a reflection of the faculty’s schedule limitations and residents’ restricted access to the ultrasound probes.

In addition, the data contains very limited numbers of Obstetrics/Gynecology (OB/GYN) and trauma (Extended Focused Assessment with Sonography in Trauma, or eFAST) scans—indications, where ultrasound is generally found to be very useful—which was likely multifactorial. First, faculty schedule limitations and the hospital’s request that faculty lead the departments of Internal Medicine and Pediatrics resulted in less availability to scan OB/GYN and trauma patients. Adding to the paucity of eFAST scans was the fact that the hospital’s Emergency Department referred all trauma patients to the region’s tertiary center, as they lacked a surgeon capable of managing severe trauma cases, so these patients were not admitted to our facility. Likely exacerbating the paucity of OB/GYN scans was the fact that residents on the OB/GYN spent most of their time performing surgical obstetric cases, with most non-operative deliveries being managed solely by midwives. Since our program’s residents were not spending as much time on Labor & Delivery, they did not contact faculty regularly for POCUS scans there. This asymmetry in scan types may have skewed the survey results. Adjustment of faculty schedules—or provision of additional POCUS faculty and probes—to ensure consistent device availability and appropriate supervision in all patient care settings would improve this imbalance.

Over the course of the study, IVC scans were likely relied upon too heavily and sometimes performed as stand-alone scans, which may have limited diagnostic reliability [14, 16]. Future studies should utilize IVC scanning only in conjunction with cardiac [20] and/or pulmonary scanning [11] and only for evidence-based indications.

The POCUS scans in this study were performed exclusively with two handheld devices, which may have reduced image quality as compared to larger, less portable ultrasound machines. Ideally, future studies would utilize more sophisticated ultrasound equipment. Utilizing residency faculty—who are intimately involved in the team’s patient care—as the POCUS experts may have introduced a level of bias in image interpretation. Future studies should utilize an external, unbiased POCUS expert. Finally, data analysis was performed using only descriptive statistics for this study. Future studies of this type would benefit from more robust data collection and use of inferential statistics, so that any differences could be evaluated for statistical significance and further inferences made.

### Conclusion and recommendations

This study suggests that resident physicians in LMICs perceive POCUS to be very useful in their medical decision-making for whom they deemed a POCUS scan necessary. Though this study’s sample is small, these data may support the advancement of POCUS education in residency programs throughout LMICs, which may be an ideal strategy to promote widespread utilization of POCUS in low-resource settings globally. Further analytical studies with larger sample sizes which evaluate impact using patient-level indicators will be necessary to further characterize the effect of POCUS education in residency programs in LMICs.

## Data Availability

The data sets used and/or analyzed during the current study are available from the corresponding author on reasonable request.

## Abbreviations

AAA: Abdominal aortic aneurysm
BLUE: Bedside lung ultrasound in emergency
CHF: Congestive heart failure
COVID: Coronavirus disease 2019
CVP: Central venous pressure
DVT: Deep venous thrombosis
ED: Emergency department
eFAST: Extended focused assessment with sonography in trauma
FASH: Focused Assessment with Sonography for HIV-associated tuberculosis
GB: Gallbladder
GE: General Electric
GUSI: Global Ultrasound Institute
IRB: Institutional review board
IVC: Inferior vena cava
L&D: Labor and delivery
LMICs: Low- and middle-income countries
MSK: Musculoskeletal
N/A: Not applicable
OB/GYN: Obstetrics and Gynecology
OSCE: Objective structured clinical examination
POCUS: Point-of-care ultrasound
RUSH: Rapid ultrasound for shock and hypotension
UNZA: University of Zambia
UNZAREC: University of Zambia Biomedical Research Ethics Committee
